# Liraglutide Pretreatment Does Not Improve Acute Doxorubicin-Induced Cardiotoxicity in Rats

**DOI:** 10.3390/ijms25115833

**Published:** 2024-05-27

**Authors:** Carolina R. Tonon, Marina G. Monte, Paola S. Balin, Anderson S. S. Fujimori, Ana Paula D. Ribeiro, Natália F. Ferreira, Nayane M. Vieira, Ronny P. Cabral, Marina P. Okoshi, Katashi Okoshi, Leonardo A. M. Zornoff, Marcos F. Minicucci, Sergio A. R. Paiva, Mariana J. Gomes, Bertha F. Polegato

**Affiliations:** 1 Department of Internal Medicine, Botucatu Medical School, São Paulo State University (UNESP), Botucatu 18618-687, SP, Brazil; marina.monte@unesp.br (M.G.M.); paola.balin@unesp.br (P.S.B.); seiji.fujimori@unesp.br (A.S.S.F.); ana.d.ribeiro@unesp.br (A.P.D.R.); natalia.fernanda@unesp.br (N.F.F.); n.vieira@unesp.br (N.M.V.); ronny.cabral@unesp.br (R.P.C.); marina.okoshi@unesp.br (M.P.O.); katashi.okoshi@unesp.br (K.O.); leonardo.zornoff@unesp.br (L.A.M.Z.); marcos.minicucci@unesp.br (M.F.M.); sergio.paiva@unesp.br (S.A.R.P.); bertha.polegato@unesp.br (B.F.P.); 2 Department of Kinesiology and Sport Management, Texas A&M University, College Station, TX 77843, USA; m.janinigomes@tamu.edu

**Keywords:** doxorubicin, heart failure, acute cardiotoxicity

## Abstract

Doxorubicin is an effective drug for cancer treatment; however, cardiotoxicity limits its use. Cardiotoxicity pathophysiology is multifactorial. GLP-1 analogues have been shown to reduce oxidative stress and inflammation. In this study, we evaluated the effect of pretreatment with liraglutide on doxorubicin-induced acute cardiotoxicity. A total of 60 male Wistar rats were allocated into four groups: Control (C), Doxorubicin (D), Liraglutide (L), and Doxorubicin + Liraglutide (DL). L and DL received subcutaneous injection of liraglutide 0.6 mg/kg daily, while C and D received saline for 2 weeks. Afterwards, D and DL received a single intraperitoneal injection of doxorubicin 20 mg/kg; C and L received an injection of saline. Forty-eight hours after doxorubicin administration, the rats were subjected to echocardiogram, isolated heart functional study, and euthanasia. Liraglutide-treated rats ingested significantly less food and gained less body weight than animals that did not receive the drug. Rats lost weight after doxorubicin injection. At echocardiogram and isolated heart study, doxorubicin-treated rats had systolic and diastolic function impairment. Myocardial catalase activity was statistically higher in doxorubicin-treated rats. Myocardial protein expression of tumor necrosis factor alpha (TNF-α), phosphorylated nuclear factor-κB (p-NFκB), troponin T, and B-cell lymphoma 2 (Bcl-2) was significantly lower, and the total NFκB/p-NFκB ratio and TLR-4 higher in doxorubicin-treated rats. Myocardial expression of OPA-1, MFN-2, DRP-1, and topoisomerase 2β did not differ between groups (*p* > 0.05). In conclusion, doxorubicin-induced cardiotoxicity is accompanied by decreased Bcl-2 and phosphorylated NFκB and increased catalase activity and TLR-4 expression. Liraglutide failed to improve acute doxorubicin-induced cardiotoxicity in rats.

## 1. Introduction

Cancer is one of the main causes of morbidity and mortality in the world and represents a major public health problem. Doxorubicin is a chemotherapy drug which has been used for many years in treating various types of cancer. Despite its extensive use, doxorubicin has many collateral effects, such as cardiotoxicity, which can lead to heart failure—the most severe side effect. Doxorubicin-induced heart failure presents a high mortality rate, with a 1-year risk of 15–30% and a 5-year risk of up to 75% in specific populations [1,2].

Several mechanisms seem to be involved in its cardiotoxicity pathophysiology, including direct damage to DNA by topoisomerase 2 inhibition, oxidative stress, activation of inflammatory pathways, alteration in intracellular calcium transient, inhibition of muscle protein related genes, mitochondrial dysfunction, and activation of cell death pathways [3,4,5,6,7].

Cardiotoxicity severity can limit doxorubicin administration and treatment; it is therefore important to identify new strategies to attenuate doxorubicin-induced cardiotoxicity.

Liraglutide, a glucagon-like peptide type 1 (GLP-1) analogue, is used in treating type 2 diabetes and obesity. It acts through G proteins linked to receptors on pancreatic beta cells, which stimulate insulin production and inhibit glucagon production, improving hyperglycemia and reducing gastric emptying. In addition, liraglutide appears to block lipid peroxidation and reduce inflammatory cytokines in a cerebral ischemia model [8]. The effects of GLP-1 analogues on myocardial metabolism remain uncertain. In experimental doxorubicin-induced cardiotoxicity, liraglutide reduced serum malondialdehyde (MDA) concentrations, a marker of oxidative damage to lipids, and tumor necrosis factor alpha (TNF-α) in rats [9]. Exenatide, also a GLP-1 analogue, decreased apoptosis and increased autophagy in the presence of doxorubicin in cultured cardiomyocytes and improved left ventricular ejection fraction in doxorubicin-treated rats [10].

In clinical studies, GLP-1 analogues reduced stroke and myocardial infarction in high-risk patients with diabetes [11], reduced cardiovascular events in diabetic patients with a previous myocardial infarction [12], decreased hospitalization in heart failure patients [13], improved diastolic function in diabetic patients [14], and reduced physical limitation symptoms and hospitalization in patients with heart failure with preserved ejection fraction and obesity [15].

In this study, we hypothesized that liraglutide protects cardiomyocytes of doxorubicin-treated rats by reducing inflammation, oxidative stress, and apoptosis. Therefore, the aim of our study was to evaluate the influence of liraglutide on morphological and functional cardiac parameters, oxidative stress, inflammation, mitochondrial biogenesis, and apoptosis in acute doxorubicin-induced cardiotoxicity in rats.

## 2. Results

### 2.1. Body Weight, Food Ingestion, and Morphological Characteristics

**Figure 1:**
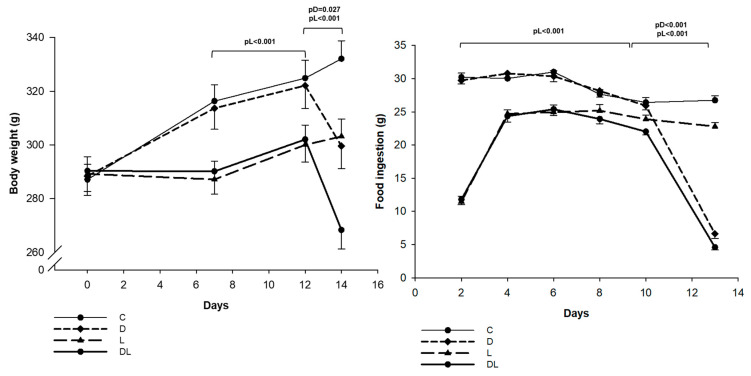
Body weight and food ingestion. The area under the curve (AUC) was compared between the groups in the periods D1–D7, D7–D12, and D12–D14 for body weight and D1–D9 and D9–D14 for food ingestion. C: control (*n* = 15); L: liraglutide (*n* = 15); D: doxorubicin (*n* = 15); DL: doxorubicin + liraglutide (*n* = 15). Data are expressed as means ± standard error. Generalized linear model (GLM); pD: *p*-value for doxorubicin effect; pL: *p*-value for liraglutide effect; pDxL: *p*-value for the interaction between doxorubicin and liraglutide.

Body weight did not differ between groups at the beginning of the experiment (C 287 ± 22; D 288 ± 29; L 289 ± 24; DL 290 ± 31 g; pDxL = 0.989; pL = 0.766; pD = 0.86). The area under the curve (AUC) of body weight did not differ between groups during the first seven days of the experiment and was smaller in the rats receiving liraglutide than those not receiving this drug during the second week. After doxorubicin administration, rats presented marked weight loss, and the AUC of this period was markedly reduced in animals that received doxorubicin (Figure 1). Animals receiving liraglutide ingested less food until D9 than the rats not receiving this drug. After doxorubicin administration, animals that received doxorubicin had significant lower food intake than those not receiving this medication (Figure 1). LV weight normalized to body weight (C 2.07 ± 0.29; D 2.02 ± 0.26; L 1.92 ± 0.23; DL 2.04 ± 0.24 g; pDxL = 0.112; pL = 0.818; pD = 0.308) did not differ between groups.

### 2.2. Echocardiography

**Figure 2:**
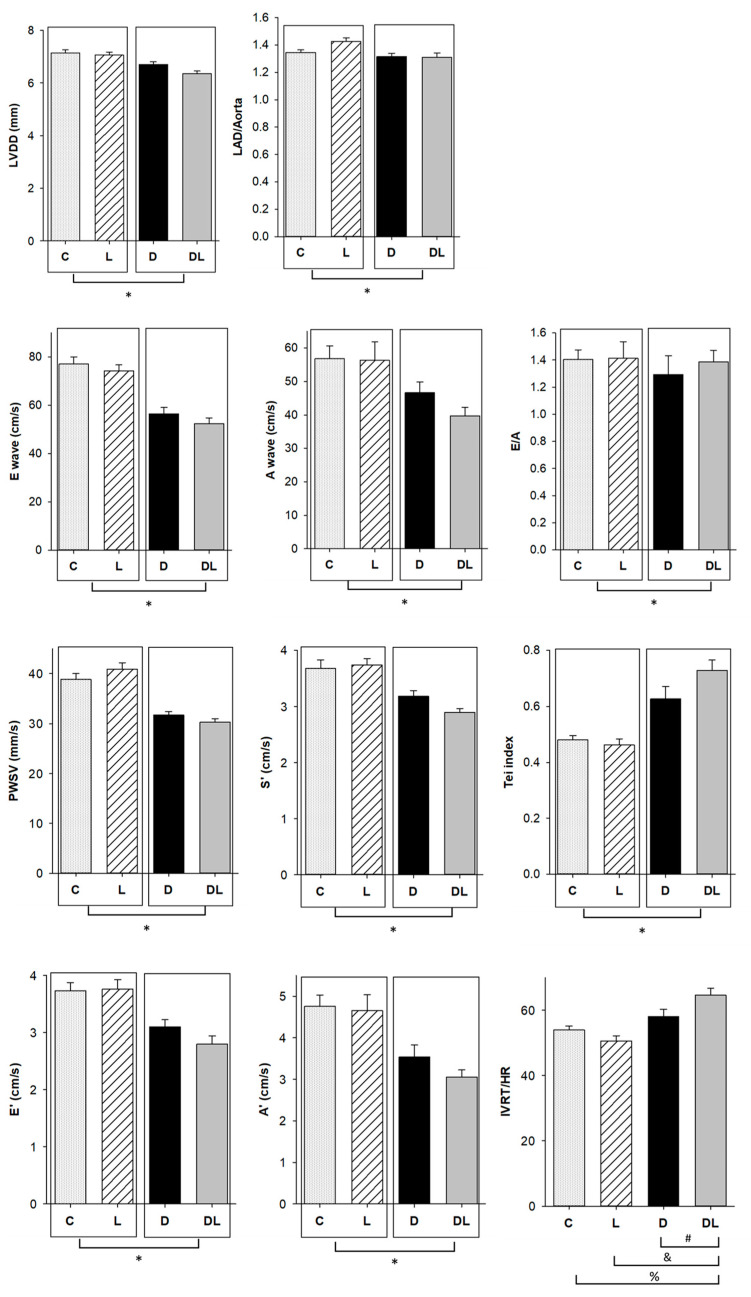
Echocardiographic structural and functional variables. C: control (*n* = 15); L: liraglutide (*n* = 15); D: doxorubicin (*n* = 15); DL: doxorubicin + liraglutide (*n* = 15); LVDD: left ventricle diastolic diameter; LAD: left atrial diameter; Aorta: aorta dimension; E: peak velocity of early ventricular filling; A: peak velocity of transmitral flow during atrial contraction; PWSV: posterior wall shortening velocity; IVRT: isovolumetric relaxation time; E’: average of mitral ring displacement of the lateral and septal walls during initial diastole in tissue Doppler image (TDI); A’: average of mitral ring displacement of the lateral and septal wall during late diastole in TDI; S’: average of mitral ring displacement of the lateral and septal wall during systole in TDI. Data are expressed as means ± standard error. Generalized linear model (GLM) or ANCOVA; * *p* < 0.05 for dox factor; % *p* < 0.05 vs. C; & *p* < 0.05 vs. L; # *p* < 0.05 vs. D.

Complete echocardiographic data are shown in Table S1 of the Supplementary Materials. Regarding heart structural variables, rats treated with doxorubicin had a lower LV diastolic diameter (LVDD) and left atrial diameter/aorta diameter ratio than those not receiving the drug, which could characterize diastolic function impairment on doxorubicin-treated animals. Diastolic function was assessed by the parameters E, A, E/A, E’, and A’ waves, which were lower in rats treated with doxorubicin. To evaluate systolic function, we analyzed S’ wave and posterior wall shortening velocity (PWSV), which were lower in animals treated with doxorubicin. The Tei index allows a combined evaluation of systolic and diastolic function. The Tei index, higher in the rats that received doxorubicin compared to those not receiving the drug, suggested impaired systolic and diastolic function in doxorubicin-treated animals. Isovolumetric relaxation time in absolute and normalized to heart rate values was higher in DL than in the C, D, and L groups, suggesting diastolic impairment in the DL group (Figure 2).

### 2.3. Isolated Heart Study

**Figure 3:**
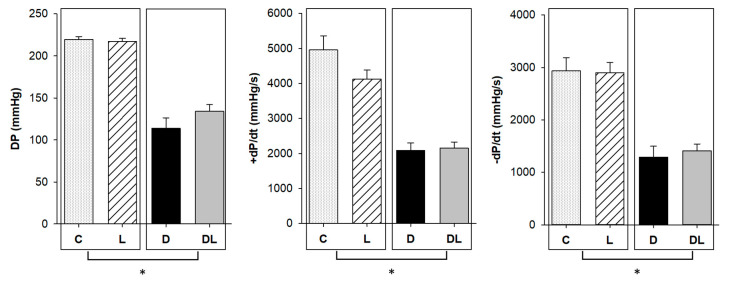
Isolated heart preparation. C: control (*n* = 5); L: liraglutide (*n* = 6); D: doxorubicin (*n* = 6); DL: doxorubicin + liraglutide (*n* = 5). DP: maximum developed systolic pressure; +dP/dt: maximum left ventricular (LV) pressure development rate; −dP/dt: maximum LV pressure decrease rate. Data are expressed as means ± standard error. Generalized linear model (GLM). * *p* < 0.05 for dox factor.

**Figure 4:**
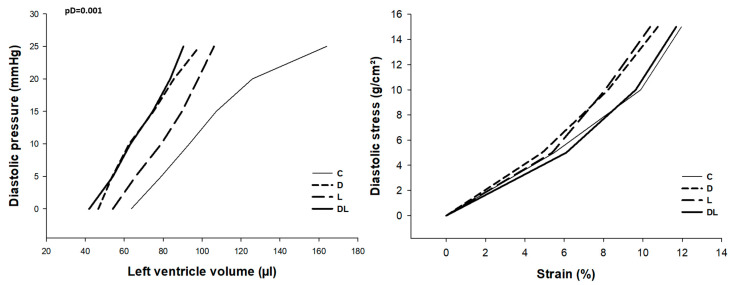
Diastolic pressure–volume curve and stress–strain relationship (AUC). C: control; D: doxorubicin; L: liraglutide; DL: doxorubicin + liraglutide. Data are expressed as means. Generalized linear model (GLM); pD: *p*-value for doxorubicin effect; *p*-value for liraglutide effect > 0.05; *p*-value for the interaction between doxorubicin and liraglutide > 0.05.

Ex vivo cardiac function was evaluated in isolated heart preparations, which allows assessment of cardiac functional parameters without humoral, neural, or hemodynamic influence. The doxorubicin-treated rats had lower maximum developed systolic pressure, +dP/dt, and −dP/dt than animals not receiving the drug, suggesting an impairment in ex vivo systolic and diastolic function (Figure 3). Ventricular compliance (Figure 4), determined by the area under the pressure–volume curve, was lower in doxorubicin-treated rats than in animals not receiving the medication. The strain–stress relationship did not differ between groups.

### 2.4. Histology

**Figure 5:**
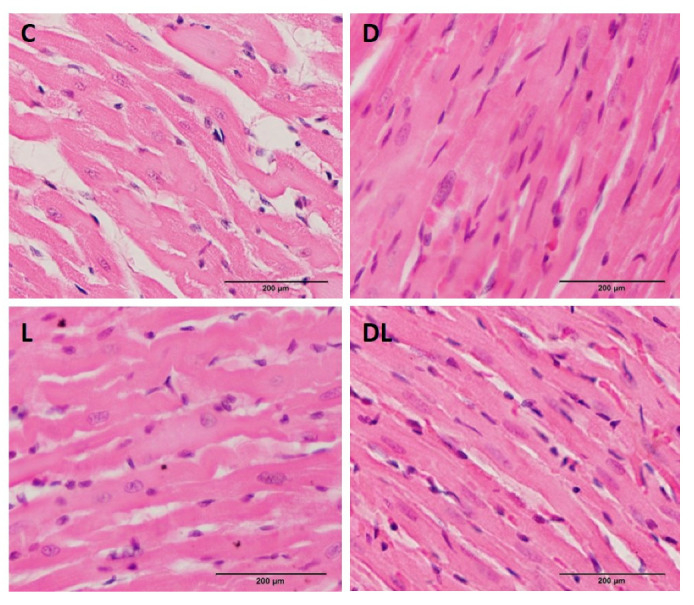
Myocardial histology. Representative longitudinal sections of the myocardium stained with hematoxylin and eosin. C: control (*n* = 7); L: liraglutide (*n* = 8); D: doxorubicin (*n* = 8); DL: doxorubicin + liraglutide (*n* = 8).

We did not observe qualitative changes in myocardial morphology (Figure 5). Since this is an acute model of cardiotoxicity, marked histological changes were not expected.

### 2.5. Oxidative Stress In Situ (DHE)

**Figure 6:**
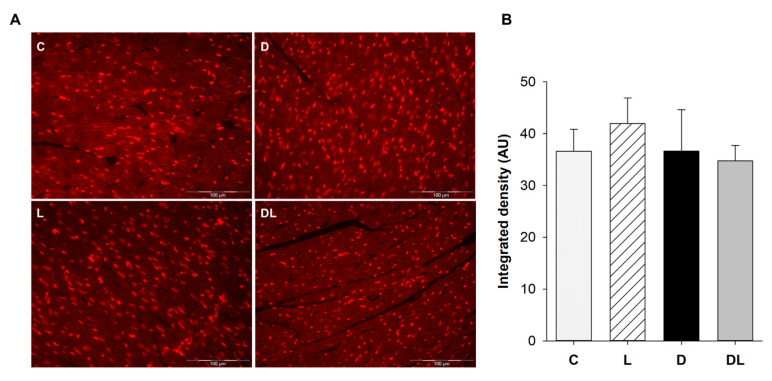
In situ oxidative stress evaluation. C: control (*n* = 5); L: liraglutide (*n* = 4); D: doxorubicin (*n* = 5); DL: doxorubicin + liraglutide (*n* = 5). (**A**) Representative myocardial sections stained with dihydroethidium to identify reactive oxygen species in the nuclei. The analysis was performed under a fluorescence microscope at 40× magnification. (**B**) Integrated density of DHE staining. Data are expressed as means ± standard error.

The in situ production of reactive oxygen species was evaluated by immunofluorescence staining with dihydroethidium (DHE) in paraffin-fixed myocardium. The integrated density was measured by the software ImageJ 1.53 (free access). We did not observe differences between groups (Figure 6).

### 2.6. Oxidative Stress and Antioxidant Enzymes

Lipid damage occurs through peroxidation, which increases MDA concentration, and protein damage occurs through oxidative carbonylation. Myocardial concentration of MDA and protein carbonylation did not differ between groups. Catalase and superoxide dismutase (SOD) are antioxidant enzymes. Catalase activity was higher in rats treated with doxorubicin than in animals not receiving this drug (Table 1). Superoxide dismutase activity did not differ between groups.

### 2.7. Western Blot

Since doxorubicin-induced cardiotoxicity is multifactorial, we evaluated proteins related to inflammation, DNA transcription, apoptosis, and mitochondrial biogenesis by Western blot.

**Figure 7:**
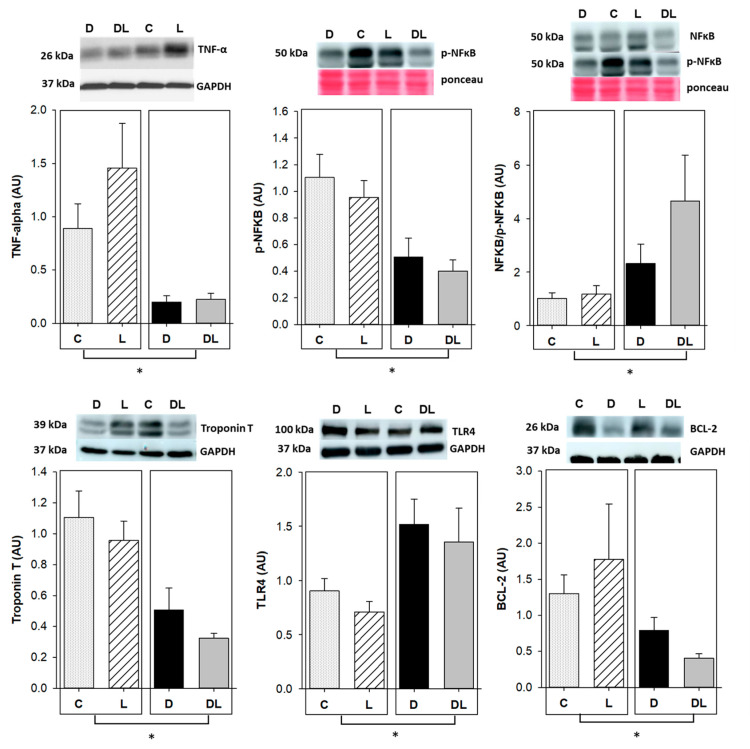
Protein expression by Western blot. Sample size: 5–7. TNF-α: tumor necrosis factor alpha; NFκB: nuclear factor kappa B; p-NFκB: phosphorylated nuclear factor kappa B; TLR-4: toll-like receptor type 4; BCL-2: B2 cell lymphoma; GAPDH: glyceraldehyde 3-phosphate dehydrogenase; C: control; D: doxorubicin; L: liraglutide; DL: doxorubicin + liraglutide; AU: arbitrary unit. Proteins were normalized by GAPDH, except for NFκB and p-NFκB, which were normalized by endogenous total protein using Ponceau staining. IL-10 and TLR4 were performed in the same membrane. Data are expressed as means ± standard error. Generalized linear model (GLM); * *p* < 0.05 for dox factor.

The inflammation-related proteins TNF-α and NFκB were assessed in the myocardium. TNF-α and phosphorylated NFκB expression was lower in doxorubicin-treated rats than in those not receiving the drug (Figure 7). Toll-like receptor 4 (TLR4) is a receptor that responds to endogenous and exogenous signals, triggering the release of inflammatory mediators. TLR4 expression was higher in animals treated with doxorubicin. We also evaluated troponin T, a constituent of myocardial proteins, which was lower in the doxorubicin-treated rats, suggesting heart damage. BCL-2, an anti-apoptotic protein, was lower in doxorubicin-treated animals, suggesting a pro-apoptotic environment.

We next evaluated the topoisomerase 2β, an enzyme that acts on DNA transcription, IL-10, an anti-inflammatory mediator, and MFN-2, DRP-1, and OPA-1, which are proteins related to mitochondrial biogenesis. The expression of these proteins did not differ between groups (Supplementary Materials, Figure S1).

## 3. Discussion

Doxorubicin is one of the most studied chemotherapy drugs because of its high effectiveness in treating several types of cancer, and especially, in reason of the severe side effects caused in systems such as the heart [16,17,18,19], intestines [20,21,22], kidney [23,24,25,26], liver [27,28], skeletal muscles [29,30,31,32], and bone marrow [33,34]. The heart is the most studied organ due to its potential development of heart failure, a chronic, limiting, and high-mortality condition [35]. There are no specific drugs to treat or attenuate doxorubicin’s side effects and several studies are being performed to identify a drug that could be used for this purpose.

Liraglutide is a relatively new drug approved for the treatment of diabetes and obesity. Recent studies have shown potential for its use in situations where inflammation, oxidative stress, and apoptosis are present. Several authors have shown reduced lipid peroxidation, reduced NFκB, TNF-α, IL-1, IL-6, nitrous oxide production, apoptosis, and cardiac collagen deposition with the use of GLP-1 analogues in some experimental models of disease [9,10,36,37,38,39,40,41,42,43,44]. In our study, we investigated the role of liraglutide on doxorubicin-induced cardiac toxicity.

As expected, chow ingestion and body weight were reduced after starting liraglutide treatment, which can be explained by the main effect of the drug, which is to decrease gastric emptying and reduce appetite [45,46]. However, approximately 7 days after initiating liraglutide, rats started to gain body weight, suggesting adaptation to the drug, as observed by other researchers [47,48,49].

To evaluate cardiotoxicity, we assessed cardiac function by echocardiogram, which showed impairment of diastolic and systolic function in rats that received doxorubicin, as previously shown in other studies [50,51,52,53,54,55,56]. Interestingly, we also observed higher IVRT in DL than D, which may suggest an impairment in diastolic function when both drugs are administered together. Some morphological and functional echocardiographic parameters may have been influenced by the decrease in body weight and hypovolemia due to doxorubicin-induced dehydration [51]. In order to minimize this influence, we evaluated isolated heart preparations, which allowed for the assessment of cardiac functional parameters without humoral, neural, or hemodynamic influence [57]. Therefore, as systolic and diastolic dysfunction was also observed in the isolated heart study, we can confidently state that doxorubicin induced acute cardiotoxicity. Additionally, we observed a lower myocardial concentration of troponin. This heart-specific regulatory protein controls the calcium interaction between actin and myosin, responsible for muscle contraction [58]. Change in troponin levels is a characteristic marker for cardiac damage and its rise in serum is widely used in clinical practice to diagnose cardiac injury. Previous studies have shown that troponin T was reduced in cardiac tissue, probably because it was released into the circulation after myocardial damage [59,60,61,62,63].

Clinical studies have shown improved cardiovascular outcomes, including reduced hospitalization and heart failure symptoms in patients with heart failure, stroke, or diabetes who received GLP-1 analogues; however, the mechanisms involved in these beneficial effects are not clear [11,12,13,14,15]. Despite the potential benefits suggested by clinical studies, liraglutide did not attenuate cardiac dysfunction in our study.

To the best of our knowledge, only two experimental studies have evaluated the use of liraglutide in doxorubicin-induced cardiotoxicity. Abbas et al. administered doxorubicin (1.25 mg/kg IP, 4 days a week for 4 weeks) followed by liraglutide (0.1 mg/kg IP, daily for 4 weeks) to rats. Doxorubicin induced cardiac damage through oxidative stress, inflammation, and apoptosis, which were attenuated by liraglutide [9]. This study differs from ours due to the chronic doxorubicin administration, which may have intensified doxorubicin toxicity; furthermore, liraglutide was administered after doxorubicin and for a more prolonged time. Taşkıran et al. showed a reduction in myocardial caspase 3, MDA, and TNF-α and in serum troponin T in rats chronically treated with doxorubicin (6 doses of 2.5 mg/kg/day) and liraglutide (1.8 mg/kg/day IP for 15 days) [64]. The above studies used a chronic doxorubicin administration protocol, suggesting that the effects of liraglutide on doxorubicin-induced cardiotoxicity may differ depending on whether the toxicity model is acute or chronic.

Our study used a protocol with a single doxorubicin dose [50,54,65] because it causes less rat suffering and saves research resources due to a shorter experimental period. This is a recognized cardiotoxicity model in which inflammation and myocardial damage can be found a few hours after doxorubicin infusion [50,51,52,53,55,66]. In our study, cardiac dysfunction was present 48 h after the drug administration.

It is well known that inflammation plays a role in cardiotoxicity, being important in the development, propagation, and maintenance of acute and chronic heart failure [67]. Activation of inflammatory pathways may be beneficial in tissue damage by promoting tissue repair [68]. However, an accentuated response may impact cardiac function through deleterious remodeling and a negative inotropic effect [69]. Inflammation in heart failure is activated and maintained by pattern recognition receptors, which include the toll-like receptors (TLRs) TLR-2, TRL-3, and TRL-4 [70]. Activation of these receptors stimulates NFκB, a transcription factor that controls the expression of genes that regulate immune response, cell growth, and proliferation [71], including pro-inflammatory cytokines such as TNF-α.

Increased TLR-4 expression was observed in the hearts of rats chronically treated with doxorubicin [72,73] and doxorubicin-induced cardiotoxicity was attenuated in TLR-4 knockout mice [74], suggesting that this signaling pathway is activated in this context. Accordingly, in our study, TLR-4 expression was higher in the doxorubicin-treated rats, reinforcing the activation of this receptor in this pathological condition.

TNF-α is a cytokine mainly produced by activated macrophages, T-lymphocytes, and natural killer cells, whose effects are mediated by two receptors, TNFR1 and TNFR2, which trigger other cytokines. It is classically considered that TNF-α activation of TNFR1 is deleterious, while TNFR2 is beneficial [68]. TNF-α expression was lower in our doxorubicin-treated rats. This result is in contrast with heart failure studies that have shown increased serum TNF-α in individuals or experimental animals [75,76,77,78]. Our results may reflect a different laboratorial approach as we evaluated myocardial TNF-α expression using Western blot while most previous studies assessed TNF-α serum concentration using ELISA. Additionally, by causing myelotoxicity, doxorubicin may alter the shape and function of white blood cells and affect the production and release of TNF-α by these cells, which may explain our finding [33,34].

Anti-inflammatory cytokines, such as IL-10, also play a role in cardiac disease by modulating the deleterious effects of exacerbated inflammation [79]. Reduced IL-10 serum concentration or gene expression was observed in rodents treated both acutely and chronically with doxorubicin [79,80,81]. These results differ from our study, as myocardial IL-10 expression did not differ between groups.

Total NFκB expression did not differ between groups, whereas p-NFκB was lower in doxorubicin-treated animals, which consequently led to an increased NFκB/p-NFκB ratio. Increased total NFκB myocardial expression was observed by immunohistochemistry in rats with acute doxorubicin-induced cardiotoxicity [82]. Also, increased NFκB gene expression was found in the kidney after chronic doxorubicin-induced nephrotoxicity [83]. Phosphorylation of NFκB subunits is a complex and not completely understood process that either enhances or downregulates the transcription of genes that affect cellular responses to several physiological processes, including inflammation and oxidative stress [84]. Oxidative stress is characterized by an imbalance between reactive oxygen species (ROS) and reactive nitrogen species (RNS) production and the action of antioxidant enzymes [85,86]. High ROS or RNS levels may activate cytotoxic signaling, leading to cellular damage, including cell membrane lipids, proteins, and DNA, mitochondrial dysfunction, NFκB activation, and intracellular calcium homeostasis dysregulation [87].

Lipid damage occurs through peroxidation, which increases MDA concentration and protein damage through oxidative carbonylation [88]. MDA concentration, protein carbonylation, and DHE in situ production of reactive oxygen species did not differ between groups in our study. DHE is a probe for a superoxide anion, a short-lived radical generated by active dehydrogenases in the presence of oxidative substrates. The fact that we evaluated DHE in fixed samples is a limitation of this method. Our results contrast with other studies showing that MDA concentration and protein carbonylation increased after doxorubicin treatment [89,90,91,92,93]. Superoxide dismutase (SOD) activity did not differ between groups in our study, while catalase activity increased in doxorubicin-treated animals. SOD is the first enzyme in the reaction that catalyzes the dismutation of the superoxide anion to hydrogen peroxide, which is subsequently detoxified to oxygen and water by catalase. SOD does not bind to cellular membranes and is rapidly excreted by the kidneys [94], which may explain why only catalase activity was changed in our study.

Furthermore, doxorubicin has a high affinity to the mitochondrial membrane, forming an irreversible complex that changes the electron transport chain and induces more ROS production and cell damage, generating a vicious cycle [95]. Additionally, doxorubicin damage to the mitochondrial membrane can activate apoptosis, which is also an important cardiotoxicity mechanism [96].

Apoptosis is the most well characterized form of programmed cell death cardiovascular disease. Extrinsic and intrinsic apoptotic pathways activate caspases and lead to cell death. The extrinsic pathway is mediated by caspases 8 and 10, and the intrinsic pathway is triggered by various extra- and intracellular stresses with cytochrome c release. Bcl-2 is an anti-apoptotic protein which prevents the release of cytochrome c [97]. Increased apoptosis has already been described in doxorubicin-induced cardiotoxicity [53,98,99,100,101]. In our study, we observed reduced Bcl-2 expression in doxorubicin-treated animals, which may reinforce a pro-apoptotic environment [89,102].

Some more recent studies have shown that doxorubicin alters mitochondrial biogenesis [103,104]. Under stress, mitochondria undergo two processes called fusion and fission, which are required for optimal cell function and survival. In fusion, two mitochondria join at the outer and inner membrane interfaces, allowing for an exchange of gene products and metabolites, enhancing their functional capacity. Mitochondrial fission is required to eliminate damaged mitochondria [105]. Fusion is controlled by proteins OPA1, MFN1, and MFN2 and fission is mediated by DRP-1 and Fis1. In vitro studies have shown a reduction in mitochondrial fusion proteins (MFN1, MFN2, OPA1) and an increase in fission protein DRP1 in cardiomyocytes from neonatal rats treated with doxorubicin [106]. Despite that, no differences in MFN2, OPA1, and DRP1 expression were observed in our groups.

The fact that we did not observe myocardial histological changes suggests that there was not enough time for them to develop. Most studies that showed cardiac histological alterations were performed under chronic doxorubicin protocols [107]. Zhu et al. observed that histological changes can only be found after 7 days from doxorubicin administration [108]. In our study, we evaluated inflammation, oxidative stress, apoptosis, and mitochondrial dynamics in rats treated with doxorubicin. However, as other pathways may be involved in doxorubicin-induced cardiotoxicity, additional studies are needed to establish the effects of liraglutide on acute toxicity.

## 4. Materials and Methods

### 4.1. Study Design

**Figure 8:**
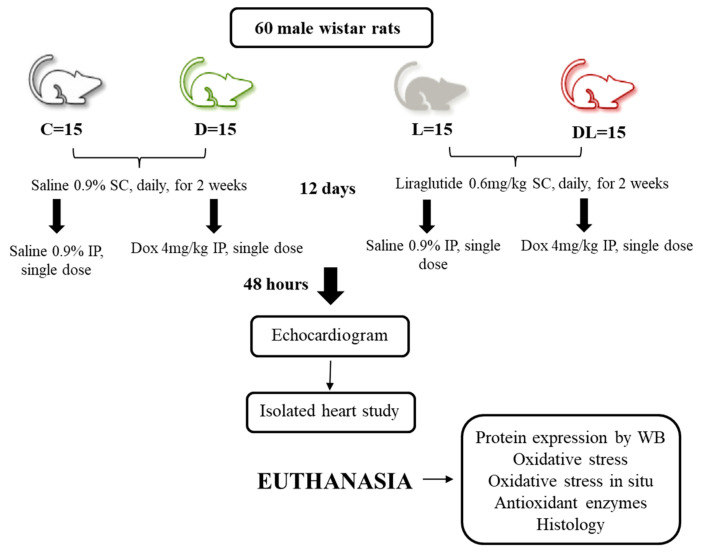
Experimental design. C: control; D: doxorubicin; L: liraglutide; DL: doxorubicin + liraglutide; SC: subcutaneous; IP: intraperitoneal; Dox: doxorubicin; WB: Western blot.

This study was registered and approved by the Ethics Committee of Sao Paulo State University, UNESP, Botucatu, under protocol No. 1285/2019, and was performed in accordance with the National Council for the Control of Animal Experimentation standards and ARRIVE guidelines. Sixty male Wistar rats weighing between 250 and 300 g were randomly allocated into four groups of 15 rats each: control (C), doxorubicin (D), liraglutide (L), and doxorubicin + liraglutide (DL). The rats were kept in a controlled environment with 12 h light/dark cycles at 23 ± 2 °C and had free access to water and regular chow. L and DL groups received 0.6 mg/kg liraglutide subcutaneously daily for 14 days while C and D groups received equivalent volumes of saline. On the 12th day of the experiment, D and DL groups received a single intraperitoneal (IP) dose of doxorubicin, 20 mg/kg, and C and L groups received a single equivalent volume IP dose of saline (Figure 8). Chow intake was measured throughout the experiment as the difference between the daily offered amount and amount remaining in the cages. Animal weight was measured at the beginning of the experiment and on the 7th, 12th, and 14th days. Liraglutide was acquired from Novo Nordisk, Brazil, and doxorubicin from Eurofarma, Brazil. At the end of the experiment, rats were anesthetized with xylazine (10 mg/kg IP) and ketamine (50 mg/kg IP) and subjected to echocardiogram. Six rats per group were then submitted to isolated heart study after an extra dose of thiopental (80 mg/kg IP). The remaining rats were euthanized after an excessive dose of thiopental (120 mg/kg IP). After blood collection, hearts were washed in fresh saline, dissected, weighed, flash frozen in liquid nitrogen, and stored at −80 °C. Blood was centrifuged at 1300 relative centrifugal force (RCF) for 10 min at 21 °C; serum was harvested and stored at −80 °C.

### 4.2. Echocardiogram

Rats were anesthetized with ketamine (50 mg/kg IP) and xylazine (1 mg/kg IP) and subjected to echocardiogram using a GE Vivid 6 from General Electric Medical Systems, Israel, with a 5.0–11.5 MHz multifrequency transducer, according to a previously described method [109,110,111]. The following structural variables were obtained by mono-dimensional analysis: left atrium diameter (LA), left ventricular (LV) diastolic and systolic diameters (LVDD and LVSD, respectively), LV diastolic and systolic posterior wall thickness (PWT), interventricular septum thickness (IVST), and aorta diameter. Systolic function was evaluated by the posterior wall shortening velocity (PWST), which is the maximum tangent of systolic movement of the LV posterior wall, and ejection fraction using the formula (LVDD^3^ − LVSD^3^)/LVDD^3^. Diastolic function was assessed by the early and late diastolic mitral inflow velocities (E and A waves, respectively), E/A ratio, E wave deceleration time (EDT), and isovolumetric relaxation time (IVRT). Combined systolic and diastolic function was assessed using the myocardial performance index, also known as the Tei index, which is the sum of the isovolumetric contraction time and IVRT, divided by the LV ejection time. Evaluation was complemented by tissue Doppler imaging (TDI) of the systolic (S’), early diastolic (E’), and late diastolic (A’) velocities of the mitral annulus. Data were used to calculate the E/E’ ratio.

### 4.3. Isolated Heart Study

The functional isolated heart study was performed as previously described [112,113,114]. Briefly, rats were anesthetized with thiopental 80 mg/kg, subjected to sternotomy, and the aorta was dissected and cannulated. Subsequently, retrograde perfusion was started with modified Krebs–Henseleit solution (NaCl 118.5 mM/L; KCl 4.69 mM/L; CaCl_2_ 2.52 mM/L; MgSO_4_ 1.16 mM/L; KH_2_PO_4_ 1.18 mM/L; glucose 5.50 mM/L; NaHCO_3_ 25.88 mM/L; and mannitol 8 mM/L). The solution was kept at 37 °C, with a perfusion pressure of 75 mmHg and a controlled O_2_ concentration. Hearts were transferred to the isolated heart study apparatus (Hugo Sachs Elektronik, March, Germany). A latex balloon connected to a pressure transducer was inserted into the LV cavity. The volume inside the balloon was increased to change diastolic pressure from 0 to 25 mmHg. After each volume variation, we recorded the diastolic and systolic LV pressures, maximum LV pressure decrease rate (–dP/dt), and maximum LV pressure development rate (+dP/dt). Myocardial diastolic stiffness in hearts with different LV weights and sizes was assessed by calculating stress (g/cm^2^) and strain (%) at the LV midwall, assuming the LV to be a thick-walled sphere. The equations used were the following:

stress = [1.36 × LVP × LVV^2/3^]/[(LVV + 0.943 × LVW)^2/3^ − LVV^2/3^]

strain = {[LVV^1/3^ + (LVV + 0.943 × LVW)^1/3^]/[V0^1/3^ + (V0 + 0.943 × LVW)^1/3^] − 1} × 100

where × is the multiplication sign, LVV is the LV volume (mL), V0 is the LV volume at an end-diastolic pressure of 0 mm Hg, LVW is the LV weight (g), and LVP is the LV end-diastolic pressure (mmHg).

### 4.4. Histology

LV cross sections were fixed in 10% buffered formalin and embedded in paraffin. Five-micron-thick sections were used to prepare histological slides, which were stained with hematoxylin and eosin. Qualitative assessment of the general morphology was performed using an optical microscope at 40× magnification, attached to a video camera, connected to a computer.

### 4.5. Oxidative Stress In Situ (DHE)

LV cross sections were fixed in 10% buffered formalin to preserve the tissue morphology and retain the antigenicity of the target molecules. Then, they were dehydrated and then embedded in paraffin. Five-micron-thick sections were used to prepare histological slides. The paraffin was removed and slides rehydrated with xylene and ethyl alcohol, as described previously [115,116]. The sections were marked with a PAP Pen (Biotium, Fremont, CA, USA) and incubated with dihydroethidium (Sigma, St. Louis, MO, USA) diluted in phosphate-buffered saline (PBS) for 30 min at 37 °C. Sections were then washed with PBS and fixed with agarose. Images were captured using a fluorescence microscope (Olympus BX51, Hamburg, Germany) at 40× magnification and analyzed using the ImageJ program (National Institutes of Health), with the quantification of the integrated density of the stained nuclei.

### 4.6. Oxidative Stress

Myocardial oxidative stress was assessed by measuring malondialdehyde, a marker of oxidative damage to lipids, and protein carbonylation, a marker of oxidative damage to proteins. Malondialdehyde was measured using the thiobarbituric acid reactive substances (TBARS) method. For quantification, 200 µL of supernatant from the homogenate of each sample was diluted in 500 µL of a solution with 0.67% thiobarbituric acid, 10% trichloroacetic acid, and 0.25 M HCl and centrifuged for 10 min at 3500 rpm. Afterwards, samples were incubated in a water bath (approximately 100 °C) for 45 min and subsequently transferred to a microplate (200 µL). Readings were performed at 532 nm and 600 nm on a Spectra Max 190 microplate reader (Molecular Devices^®^, Sunnyvale, CA, USA). For protein carbonylation analysis, 100 µL of tissue supernatant was added to 100 µL of 2,4-dinitrophenylhydrazine (DNPH). Samples were incubated for 10 min at room temperature, followed by the addition of 50 µL of NaOH 6 M, and incubated for 10 min at room temperature. Readings were carried out at 450 nm on a Spectra Max 190 microplate reader to obtain sample absorbance values and the molar extinction coefficient (22,000 M^−1^·cm^−1^).

### 4.7. Antioxidant Enzymes

Samples of approximately 100 mg of LV tissue were homogenized in a sodium phosphate buffer 0.01 M at a pH of 7.4 and centrifuged for 30 min at −4 °C; total protein in samples was quantified by the Bradford method. Superoxide dismutase (SOD) activity was measured by pyrogallol oxidation detected by spectrophotometry at 420 nm, and catalase activity was determined by hydrogen peroxide consumption detected by spectrophotometry at 240 nm.

### 4.8. Protein Expression by Western Blot

LV muscle (100 mg) was added to extraction buffer, homogenized, and centrifuged. Supernatants were collected and total protein quantified by the Bradford method. Electrophoresis was performed in acrylamide gels and proteins were transferred to nitrocellulose membranes and incubated in 5% skimmed milk. Subsequently, membranes were incubated with primary antibodies from Santa Cruz Biotechnology (Dallas, TX, USA), anti-TNF-α, total NFκB, phosphorylated NFκB, interleukin 10 (IL-10), toll-like receptor type 4 (TLR-4), troponin T, topoisomerase 2β, B-cell lymphoma 2 (BCL-2), mitofusin-2 (MFN2), dynamin-related protein (DRP-1), and optic atrophy type 1 (OPA-1) for 12 h, followed by incubation with secondary antibodies (rabbit anti-mouse, IgG, ab6728, Abcam, Cambridge, UK). Information on antibody manufacturers and dilutions is shown in the Supplementary Materials. Immunodetection was performed using chemiluminescence in the ImageQuant LAS camera imaging system (General Electrics, Schenectady, NY, USA). Images were analyzed by Gel-Pro 32 (Media Cybernetics, Rockville, MD, USA). Glyceraldehyde 3-phosphate dehydrogenase (GAPDH) or Ponceau staining (Merck, Darmstadt, Germany) was used to normalize all proteins.

### 4.9. Statistical Analyses

Variables are shown as means ± standard error. Comparisons between groups were performed by the generalized linear model (GLM), with gamma distribution, and groups were considered as independent variables. We present three *p*-values: pDxL for the interaction between doxorubicin and liraglutide, pD for the doxorubicin factor, and pL for the liraglutide factor. When there was interaction between doxorubicin and liraglutide (pDxL < 0.05), results are presented comparing groups of interest using the Bonferroni post-test. When there was no interaction between factors (pDxL > 0.05), marginal data are presented, referring to the effect of each factor separately.

Body weight and food ingestion were compared by the area under the curve (AUC) for different periods using GLM.

Functional echocardiographic variables were compared by using covariance analysis (ANCOVA) to correct the effect of heart rate on the parameter of interest. A statistical significance level of *p* < 0.05 was adopted for all analyses. We used Jamovi Version 2.3 software, with free access available at https://www.jamovi.org, accessed on 10 December 2023.

## 5. Conclusions

Doxorubicin caused acute cardiotoxicity, characterized by changes in cardiac structure and function, in proteins related to apoptosis and inflammation, and in catalase activity. Liraglutide administration failed to attenuate doxorubicin-induced cardiotoxicity in rats.

## Figures and Tables

**Table 1:** Oxidative stress.

	C (*n* = 7)	L (*n* = 8)	D (*n* = 8)	DL (*n* = 8)	pL	pD	pDxL
MDA (nmol/mg)	15.6 ± 6.9	14.0 ± 4.2	14.5 ± 2.3	16.7 ± 5.4	0.867	0.663	0.297
Protein carbonylation (nmol/mg)	32.2 ± 7.8	28.9 ± 5.8	31.7 ± 8.0	35.9 ± 11.5	0.287	0.869	0.219
SOD (U/ug·min)	8.3 ± 2.1	7.7 ± 1.3	7.9 ± 1.8	8.5 ± 2.0	0.984	0.785	0.329
CAT (pmol/mg·min)	2.5 ± 0.9	2.6 ± 0.8	4.1 ± 1.8	5.0 ± 1.7	0.328	**<0.001**	0.407

C: control; L: liraglutide; D: doxorubicin; DL: doxorubicin + liraglutide. MDA: malondialdehyde, SOD: superoxide dismutase; CAT: catalase. Data are expressed as means ± SD. Generalized linear model (GLM); pD: *p*-value for doxorubicin effect; pL: *p*-value for liraglutide effect; pDxL, *p*-value for the interaction between doxorubicin and liraglutide.

## Data Availability

Data is contained within the article and Supplementary Materials.

## Supplementary Materials

The following supporting information can be downloaded at: https://www.mdpi.com/article/10.3390/ijms25115833/s1.
