# Supplementary material for: Liraglutide Pretreatment Does Not Improve Acute Doxorubicin-Induced Cardiotoxicity in Rats
Source: Int J Mol Sci. 2024 May 27;25(11):5833. doi: 10.3390/ijms25115833 (PMC11172760; doi:10.3390/ijms25115833)
Supplement: Supplementary file 1 [file ijms-25-05833-s001.zip › ijms-2942783-supplementary.pdf]

Supplementary Materials

Table S1. Echocardiographic data.

	C (n=15)	L (n=15)	D (n=15)	DL (n=15)	pD	pL	pDxL
**HR (bpm)**	336 ± 41	365 ± 71	321 ± 46	291 ± 42**&**	0.001	0.974	**0.026**
**LVDD (mm)**	7.14 ± 0.45	7.05 ± 0.45	6.70 ± 0.42	6.36 ± 0.38	**<0.001**	**0.049**	0.233
**LVSD (mm)**	3.42 ± 0.41	3.32 ± 0.56	3.31 ± 0.39	3.15 ± 0.42	0.226	0.264	0.794
**PWT (mm)**	1.19 ± 0.05	1.18 ± 0.06	1.20 ± 0.05	1.15 ± 0.06	0.572	**0.031**	0.278
**IVST (mm)**	1.19 ± 0.04	1.18 ± 0.06	1.20 ± 0.05	1.16 ± 0.06	0.781	0.072	0.243
**Aorta (mm)**	3.70 ± 0.16	3.62 ± 0.15	3.53 ± 0.13	3.45 ± 0.11	**< 0.001**	**0.034**	0.916
**LAD (mm)**	4.99 ± 0.33	5.17 ± 0.40	4.60 ± 0.39	4.53 ± 0.51	**< 0.001**	0.732	0.168
**LAD/Aorta**	1.35 ± 0.08	1.43 ± 0.10	1.32 ± 0.09	1.31 ± 0.13	**0.007**	0.153	0.098
**E (cm/s)** $	77.2 ± 10.5	74.3 ± 7.40	56.6 ± 9.02	52.5 ± 9.57	**< 0.001**	0.180	0.825
**A (cm/s)** $	56.9 ± 13.8	56.3 ± 16.6	46.7 ± 10.4	39.8 ± 10.3	**0.010**	0.510	0.293
**E/A** $	1.40 ± 0.26	1.41 ± 0.37	1.29 ± 0.46	1.39 ± 0.34	**0.016**	0.980	0.595
**PWSV (mm/s)** $	38.9 ± 4.46	40.9 ± 5.02	31.8 ± 2.53	30.3 ± 2.90	**< 0.001**	0.788	0.228
**IVRT (ms)**	22.9 ± 2.82	20.9 ± 3.72	25.3 ± 4.04	29.6 ± 4.59***#&**	< 0.001	0.257	**0.002**
**IVRT/HR**	53.9 ± 4.87	50.6 ± 6.07	58.1 ± 8.94	64.7 ± 8.76***#&**	< 0.001	0.385	**0.010**
**Tei index** $	0.48± 0.06	0.46 ± 0.08	0.63 ± 0.17	0.73 ± 0.15	**<0.001**	0.241	0.146
**EF (%)** $	0.89 ± 0.02	0.89 ± 0.04	0.88 ± 0.03	0.88 ± 0.03	0.929	0.960	0.353
**S' (cm/s)** $	3.68 ± 0.59	3.74 ± 0.45	3.18 ± 0.40	2.89 ± 0.29	**< 0.001**	0.286	0.464
**E' (cm/s)** $	3.73 ± 0.50	3.76 ± 0.47	3.10 ± 0.40	2.80 ± 0.56	**<0.001**	0.330	0.193
**A' (cm/s)** $	4.75 ± 0.96	4.65 ± 1.09	3.54 ± 0.92	3.06 ± 0.66	**< 0.001**	0.431	0.456
**EDT** $	42.8 ± 5.56	43 ± 3.74	34.4 ± 8.62	42.4 ± 7.94	0.090	0.147	0.144
**E/E'** $	20.9 ± 3.33	20.2 ± 3.15	18.8 ± 4.11	19.2 ± 3.12	0.058	0.728	0.549

C: control; D: doxorubicin; L: liraglutide; DL: doxorubicin + liraglutide; HR: heart rate; LVDD: left ventricle (LV) diastolic diameter; LVSD: LV systolic diameter; PWT: end-diastolic posterior wall thickness; IVST: end-diastolic interventricular septum thickness; LAD: left atrial diameter; E: peak velocity of early ventricular filling; A: peak velocity of transmitral flow during atrial contraction; PWSV: posterior wall shortening velocity; IVRT: isovolumetric relaxation time; EF: ejection fraction; E': average of mitral ring displacement of the lateral and septal walls during initial diastole in tissue Doppler image (TDI); A': average of mitral ring displacement of the lateral and septal wall during late diastole in TDI; S': average of mitral ring displacement of the lateral and septal wall during systole in TDI; EDT: E-wave deceleration time. Data are expressed as mean ± SD. Generalized linear model (GLM); pD: p-value for doxorubicin effect; pL: p-value for liraglutide effect; pDxL: p-value for the interaction between doxorubicin and liraglutide; * different from C; # different from D; &: different from L. $ p-values were obtained by ANCOVA.

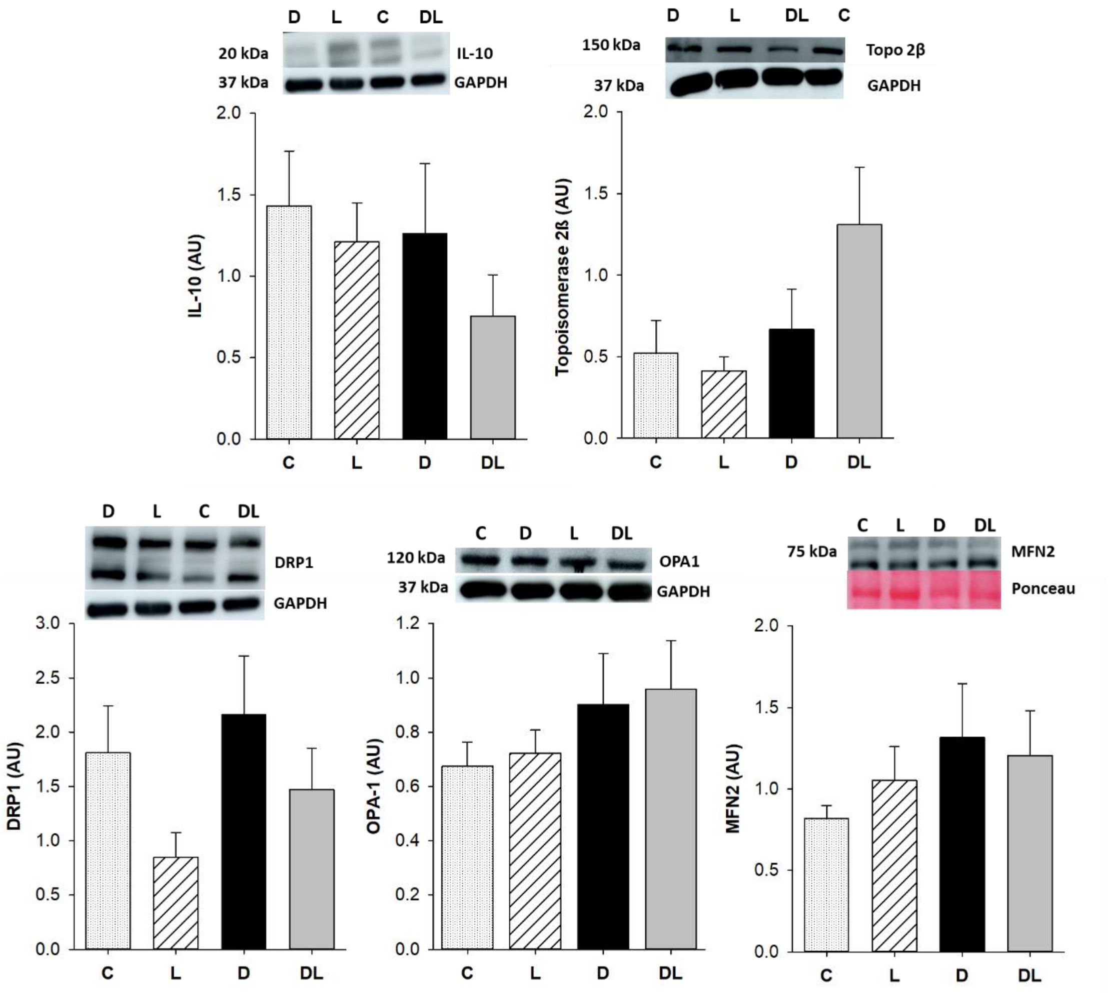

Figure S1. Protein expression by Western blot. Sample size: 5-7. IL-10: interleukin 10; MFN-2: mitofusin 2; DRP-1: dynamin-related protein; OPA-1: optic atrophy-related gene; GAPDH: glyceraldehyde 3-phosphate dehydrogenase; C: control; D: doxorubicin; L: liraglutide; DL: doxorubicin + liraglutide, AU: arbitrary units. Proteins were normalized by GAPDH, except for MFN-2 values which were normalized by endogenous total protein using Ponceau staining. Data are expressed as mean ± SD. Generalized linear model (GLM).

Antibodies information

Primary Antibody	Type	Reference	Dilution
TNF- α	Mouse monoclonal IgG	SC-52746	1:200
NFκB	Mouse monoclonal IgG	SC-8008	1:200
p-NFκB	Mouse monoclonal IgG	SC-136548	1:200
IL-10	mouse monoclonal IgG	SC-365858	1:100
TLR-4	Mouse monoclonal IgG	SC-293072	1:200
Troponin T	Mouse monoclonal IgM	SC-515899	1:100
Topoisomerase 2β	Mouse monoclonal IgG	SC-365421	1:100
BCL-2	Mouse monoclonal IgG	SC-7382	1:200

DRP-1	Mouse monoclonal IgG	SC-271583	1:100
MFN2	Mouse monoclonal IgG	SC-515647	1:100
OPA-1	Mouse monoclonal IgG	SC-393296	1:100
GAPDH	Mouse monoclonal IgG	SC-32233	1:15000
